# Supplementary material for: A qualitative exploration of Australian eyecare professional perspectives on Age-Related Macular Degeneration (AMD) care
Source: PLoS One. 2020 Feb 11;15(2):e0228858. doi: 10.1371/journal.pone.0228858 (PMC7012424; doi:10.1371/journal.pone.0228858)
Supplement: S1 Table — (DOC) [file pone.0228858.s001.doc]

**S1 Table. Semi-structured Interview Guide**

| **ID No**  **Recorder**  **Date** |
| --- |
| **1. Qualitative interview introduction**  Length: approximately 30 minutes  Topic: Gather your opinion regarding the factors that promote and prevent good AMD care. |
| **2. Consent**  Consent Form explained and signed.  Do you agree for this interview to be recorded? |
| **3. Background information**  Overview. Invite practitioner to briefly talk about experiences of AMD in their practice.  *How do AMD patients present to your practice? Do you get referrals? From who? What is a typical stage of AMD on presentation? Appropriateness of these referrals i.e. are they accurate (i.e. right diagnosis)? Are the referred at the right time? Could any aspects of referrals be improved in your opinion?* |
| **4. AMD patient journey**  From your perspective, what are some of the factors preventing people with AMD from accessing AMD care?  Invite practitioner to describe the ideal AMD patient journey.  What could be done to enable better access?  *Do you co-manage with other health providers? Who is involved? Who should be involved?*  *Factors preventing people with AMD from accessing AMD care. What could enable better access?*  *Probe about costs? Public versus private.* |
| **5. Communication with AMD patient**  From your perspective, what are some of factors preventing people with AMD from following the advice given to them by their practitioner?  Invite practitioner to describe their experience and approach to communication with AMD patients.  What more could be done to help practitioners give the right advice?  What more could be done to help AMD patients following the advice they are given?  *Describe approach. Probe about patient reaction.*  *What information or recommendations are given to patients? Ask specifically (if not mentioned) about: cures, injections, lifestyle, smoking, nutrition, exercise, Amsler grid testing, supplements.*  *Likelihood of patients following this advice. How do you assess this?*  *Ask about format of information. Any supporting documentation. Who is giving information? What is useful / not useful.*  *Do you talk about prognosis? How? Probe about words like blindness, risk, etc.*  *Role of family / friends.* |
| **6. Interview close**  Thank practitioner. Give opportunity to discuss anything else. |
