# Supplementary material for: A qualitative exploration of Australian eyecare professional perspectives on Age-Related Macular Degeneration (AMD) care
Source: PLoS One. 2020 Feb 11;15(2):e0228858. doi: 10.1371/journal.pone.0228858 (PMC7012424; doi:10.1371/journal.pone.0228858)
Supplement: S2 Table — AMD care category themes and barriers to AMD care, category of influence and rank-ordering votes across the seven optometry focus groups. (DOCX) [file pone.0228858.s002.docx]

**S2 Table. AMD care category themes and barriers to AMD care, category of influence and rank-ordering votes across the seven optometry focus groups.**

| **Category themes and associated barriers nominated across focus groups** | **Category of influence** | **No of votes** | **Score** | **Rank ordering votes across groups** | **Total score** |
| --- | --- | --- | --- | --- | --- |
| **Cost / funding**   - FG3: “Can’t afford supplements / health food. Can’t afford care (e.g. ophthalmologists, injections, transport). Cost of fundus photos” - FG1: “Supplements, imaging equipment, socio economic status” - FG2: “Lack of funding for various aspects of care (e.g. counselling); patients take up lots of chair time but no profitable – barrier to good care” - FG6: “Cost (food, supplement, consultation services)” - FG7: “Cost: medications, no public ophthalmology, injections” - FG2: “Cost of care and implications for having to wait & travel to public system” - FG5: “Cost of treatment / medication” - FG3: “PBS not for anti-VEGF for some patients” - FG7: “No health fund/medicare rebate for photos or OCT scan” - FG4: “Real and perceived cost of accessing health (e.g. nutritionist, My Aged Care)” | Structural | 12  11  8  5  4  5  4  2  1  2 | 38  34  33  19  19  14  13  7  5  3 | Rank 1: 15  Rank 2: 15  Rank 3: 7  Rank 4: 12  Rank 5: 5 | 185 |
| **Understanding / denial**   - FG3: “No functional loss – patients don’t have symptoms in early disease. Denial / fear / lack of belief. Scepticism” - FG5: “Patient denial / fear / mental state. Stigma, loss of independence, pride. Depression” - FG5: “Lack of understanding of benefits of treatment or management. Vision not improving with anti-VEGF injection – makes it hard to maintain patient compliance. Ongoing management / not a cure. Perception of optom / ophthalmol not doing anything” - FG1: “Patient underestimating the severity / seriousness of disease; people’s skewed perception of risk” - FG2: “Patients don’t understand the morbidity / the seriousness & do nothing” - FG1: “Lack of knowledge or lack of education” - FG5: “Lack of patient & carer understanding of significance of AMD diagnosis. Something they can’t see so they don’t realise seriousness” - FG2: “Patient in denial / don’t accept the diagnosis because of their age. Can’t be bothered / too hard” - FG7: “Patients don’t understand the lifetime risk / message does not get through / if they don’t have family history” - FG1: “Patients knowledge on ‘how to’ follow up” - FG4: “Scepticism / disbelief that treatment will help – no tangible benefits” - FG4: “[Patient] not informed, lack of awareness of risk factors (optoms & patients)” - FG3: “Poor understanding” - FG7: “Patient complacency – asymptomatic” - FG6: “Don’t understand injections / fear of injections” - FG1: “Fear – e.g. injections” | Patient-centered | 8  6  8  6  7  5  6  6  2  2  1  1  3  1  1  1 | 32  21  19  18  17  17  16  12  8  6  5  4  4  3  1  1 | Rank 1: 14  Rank 2: 11  Rank 3: 11  Rank 4: 9  Rank 5: 19 | 184 |
| **Access / availability of services**   - FG3: “Difficulty accessing services (e.g. transport). Living too far away. No services in regional areas” - FG5: “Location restriction to access services – e.g. regional areas. [Lack of] transport / support (family / carer) to get to appointments” - FG6: “Transport (rural a particular challenge)” - FG6: “Access to services for rural patients (no low vision services)” - FG7: “Difficulty of access / those with co-morbidities / those in nursing homes / those who have to reply on others / other co-morbid conditions are more important to the patient” - FG1: “No access to imaging equipment, geographic location” - FG6: “Larger distances in rural areas” - FG3: “Difficult to access [services / care] in nursing home” - FG3: “No access to Amsler, equipment (e.g. OCT). [No access to] practice aids. Poor access to FAF, OCT in primary care optometry” - FG7: “Long waiting time for ophthalmology appointments (more in the country) / Lack of ophthalmologists” - FG5: “Not knowing that home services are available (rehab/training)” - FG7: “Availability of practitioners who can do injections (i.e. nurse practitioners / optoms)” | Structural | 8  5  3  4  3  2  1  1  1  1  1  1 | 26  18  14  13  9  7  4  4  3  2  2  1 | Rank 1: 7  Rank 2: 7  Rank 3: 9  Rank 4: 5  Rank 5: 3 | 103 |
| **Discipline silos**   - FG2: “Other healthcare practitioners’ knowledge of optometry’s role. Lack of recognition / understanding of optometry’s role in prevention” - FG1: “Lack of optometrist communication with GP and other practitioners – patients understanding of long-term condition (ongoing practitioner communication to patient)” - FG2: “Poor / limited communication between health professionals” - FG5: “Mixed messages on supplements (from practitioners/GPs/general public)” - FG7: “Lack of co-management / shared care between optometrist and ophthalmologist” - FG5: “[Lack of] Optom / GP relationships (e.g. quit smoking)” - FG2: “Patients not told / offered all options for care (e.g. low vision, nutrition)” - FG3: “Not telling them ‘how to’ quit smoking – Refer to GP / or specialist” - FG4: “Lack of data sharing between practitioners (e.g. imaging, etc.)” - “Conflicting messages from health practitioners” - FG3: “Dietary advice not specific enough / too much like every other advice they get” - FG3: “Lack of care plan” | Clinician-centered  Structural | 9  6  4  4  2  1  1  2  1  1  1  1 | 24  15  9  6  4  3  3  3  2  1  1  1 | Rank 1: 0  Rank 2: 3  Rank 3: 11  Rank 4: 8  Rank 5: 11 | 72 |
| **Care pathway**   - FG4: “Lack of awareness of services (patients & optoms). Poor service pathway / services not accessible. Pathways not clear. Unclear to patients and practitioners. Can optoms refer directly to dietician? (pathways / referrals / care plan). Optoms not knowing what forms / helps is available to patients (e.g. taxi subsidies)” - FG2: “No coherent flowchart. No clear plan (e.g. diabetes plan). No one person responsible. No AMD care plan. Complexity and inter-relatedness of the management pathway. Correct level of access to [care]. Practitioner’s lack of awareness of services” - FG3: “Complex health system / broken” | Structural  Clinician-centered | 4  10  3 | 19  45  7 | Rank 1: 10  Rank 2: 4  Rank 3: 1  Rank 4: 0  Rank 5: 2 | 71 |
| **Co-morbidities**   - FG1: “Least of their problem – other problems (e.g. BP, sugar) more important” - FG3: “Conflicting priorities / co-morbidities” - FG6: “Age: mobility – cognitive abilities” - FG6: “Co-morbidities – e.g. dementia” - FG3: “Depression / mental health issues / dementia” - FG1: “Patient intellectual inability (e.g. dementia, aging, etc.)” - FG3: “Monthly treatment interferences with quality of life” - FG4: “Not a priority – other comorbidities” - FG5: “Other health problems/ co-morbidities (patient/carer/family)” - FG4: “Treatment / consultation fatigue (too many appointments – too many pills)” - FG4: Memory problems | Patient-centered | 6  3  2  2  2  1  1  2  1  1  1 | 25  7  6  6  6  5  5  4  3  2  1 | Rank 1: 5  Rank 2: 2  Rank 3: 10  Rank 4: 2  Rank 5: 3 | 70 |
| **Lifestyle changes difficult**   - FG1: “Habit – difficult to change – older (e.g. diet difficult to change and monitor by GP and optom)” - FG3: “Changing behaviours is hard, changing an ‘old dog’” - FG5: “Patient don’t like fish or vegan/vegetarian (will & want). Enjoy smoking / eating / not wanting to change habits. Difficult to change habits” - FG1: “No immediate improvement with changes in lifestyle” - FG7: “Stop smoking is hard / changing diet is hard – message coming from optometrist may not be heard” - FG5: “Not willing to change behaviours (e.g. diet, cigarettes, ‘I like smoking’)” - FG4: “Changing diet is hard / modifying habit is hard (willpower)” | Patient-centered | 7  6  4  2  3  2  1 | 18  16  9  8  7  6  4 | Rank 1: 0  Rank 2: 8  Rank 3: 5  Rank 4: 9  Rank 5: 3 | 68 |
| **Compliance**   - FG1: “AMD slow progression – patient not willing to do anything early on (patient not motivated when VA is still good” - FG3: “Lack of compliance (forget)” - FG3: “Lack of motivation” - FG4: “[Lack of] motivation” | Patient-centered | 7  6  2  1 | 22  18  5  3 | Rank 1: 4  Rank 2: 0  Rank 3: 5  Rank 4: 6  Rank 5: 1 | 48 |
| **Communication**   - FG1: “Poor quality of message given to or with (patients and carers)” - FG3: “Poor communication” - FG4: “Optoms don’t ask the right questions (e.g. diet / smoking)” - FG2: “Conflicting messages from health practitioners” | Clinician-centered  Patient-centered | 12  2  1  1 | 28  6  3  1 | Rank 1: 2  Rank 2: 2  Rank 3: 2  Rank 4: 4  Rank 5: 6 | 38 |
| **Underutilisation of optometry**   - FG7: “Poor GP knowledge of optometry’s role / underunitilisation of optometry in community / poor GP knowledge of eye / referral pathway / unclear referral pathway (GP → optom → ophthal but optom could triage) / GPs don’t refer like they do for diabetes” - FG5: “No regular eye checks - [using] ready mades [spectacles]” - FG5: “Patient and ophthal not knowing that rehab can happen at same time as treatment” - FG7: “Lack of knowledge of optometry’s role in the general public” - FG7: “Not having regular eye check – general public” - FG7: “Lower quality vision assessments by GPs / other health practitioners (particularly VA)” | Patient-centered  Structural | 3  2  2  1  1  1 | 12  6  5  4  3  2 | Rank 1: 1  Rank 2: 4  Rank 3: 2  Rank 4: 2  Rank 5: 1 | 32 |
| **Scope of practice**   - FG5: “Practitioners have limited knowledge; give +/- advice” - FG2: “Lack of across the board investment by optometrists to provide appropriate care (e.g. OCT, other imaging)” - FG1: “Sub-optimal diagnosis or treatment” - FG3: “Lack of trust. Lack of follow-up” - FG6: “[Practitioner] knowledge of existing services for rural patients” - FG4: “Lack of continuity of care (e.g. locum)” | Clinician-centered  Structural | 2  4  1  2  1  1 | 9  8  5  5  4  1 | Rank 1: 2  Rank 2: 4  Rank 3: 0  Rank 4: 1  Rank 5: 4 | 32 |
| **Incurable disease**   - FG3: “Lack of tangible treatment effect” - FG6: “No cure” - FG1: “No care - current treatments can’t cure” | Patient-centered | 6  2  1 | 20  2  1 | Rank 1: 2  Rank 2: 1  Rank 3: 1  Rank 4: 1  Rank 5: 4 | 23 |
| **Support**   - FG1: “Lack of carer support: patient issues can also be carer issues, should communicate to carer, patient not wanting to be a burden to carers” - FG5: “Lack of family support / more isolation” - FG3: “Lack of support (may need carer help)” | Patient-centered | 5  2  2 | 15  4  3 | Rank 1: 0  Rank 2: 3  Rank 3: 0  Rank 4: 4  Rank 5: 2 | 22 |
| **Injection**   - FG5: “Fear / trauma / pain of injections” - FG3: “Trauma of anti VEGF injections” | Patient-centered | 2  1 | 10  5 | Rank 1: 3  Rank 2: 0  Rank 3: 0  Rank 4: 0  Rank 5: 0 | 15 |
| **Time**   - FG4: “Length of consultation prevents good history taking” - FG1: “Time – patients and optoms” - FG3: “Time” | Structural | 1  1  1 | 4  3  1 | Rank 1: 0  Rank 2: 1  Rank 3: 1  Rank 4: 0  Rank 5: 1 | 8 |
| **Care guidelines**   - FG4: “Lack of clarity about best managing / standard treatment. No clear guidelines for practice” - FG5: “Lack of clear co-management guidelines” | Clinician-centered | 2  1 | 5  1 | Rank 1: 0  Rank 2: 0  Rank 3: 1  Rank 4: 1  Rank 5: 1 | 6 |

FG1 = Melbourne, VIC (Metropolitan); FG2 = Gold Coast, QLD (Metropolitan); FG3, FG4, FG5 = Sydney, NSW (Metropolitan); FG6 = Toowoomba, QLD (Regional); FG7 = Orange, NSW (Regional)
