# Supplementary material for: A qualitative exploration of Australian eyecare professional perspectives on Age-Related Macular Degeneration (AMD) care
Source: PLoS One. 2020 Feb 11;15(2):e0228858. doi: 10.1371/journal.pone.0228858 (PMC7012424; doi:10.1371/journal.pone.0228858)
Supplement: S3 Table — Barriers nominated by optometrists in at least one focus group but not selected during the “top five barriers” ranking process. (DOCX) [file pone.0228858.s003.docx]

**S3 Table. Barriers nominated by optometrists in at least one focus group but not selected during the “top five barriers” ranking process.**

| **Category themes and associated barriers nominated across focus groups** | **Category of influence** |
| --- | --- |
| **Cost / funding**   - FG3: “No medicare rebate for optometry referring direct to dietician, etc.” - FG3: “No reimbursement for giving nutrition advice” - FG3: “Pressure to ‘convert’” - FG5: “No medicare benefit / charges” - FG5: “Patient not knowing where to go to, especially if needing low cost options”   **Understanding / denial / fear**   - FG2: “Practitioners ‘play down’ the seriousness of condition” - FG2: “Stigma around being blind / visual impairment” - FG2: “Fear of losing driver’s license” - FG4: “Crazy Eddy “I’ll be fine” - FG6: “Culture ‘she’ll be right’” - FG6: “Fear (human nature)” - FG7: “Not aware of family history”   **Access / availability of services**   - FG1: “More lost to follow up in public system because of waiting list” - FG2: “Co-morbidities make it difficult to get to services” - FG2: “Transport issues (e.g. low vision patients can’t drive and often that’s when they first access low vision services)” - FG3: “No access to magnifiers / low vision aids” - FG3: “Poor access due to distance / cost / availability” - FG4: “Poor vision reduces their [patients] ability to access / follow advice” - FG4: “Transport / mobility aide may be needed” - FG6: “Poor access to home visits from low vision services”   **Discipline silos**   - FG3: “Disagreement between optometrists / ophthalmologists. Conflicting advice” - FG4: “Conflicting advice from different practitioners” - FG7: “Ophthalmologists don’t send patients back to optom”   **Care pathway**   - FG2: “Patients with wet AMD: ophthalmologists don’t consider / refer for low vision care early enough” - FG3: “Clear share-care arrangements / communication on who does what?”   **Co-morbidities**   - FG3: “Low vision aids don’t work very well in some people (cognitive, Parkinson’s, etc.)” - FG5: “Vision impairment too debilitating” - FG5: “Bad memory / forgot instructions / old / can’t read / can’t hear” - FG7: “Patients have other priorities”   **Compliance**   - FG5: “Reminders (not enough) from practitioners”   **Communication**   - FG1: “Not meeting wants or needs in the way message is delivered” - FG2: “Patients can’t remember verbal advice (should be written down)” - FG3: “Patient don’t take the handouts” - FG4: “Technological gulf – older age people less likely to use internet where resources may be located” - FG4: “Poor rapport with practitioner or practitioner has not emphasized enough” - FG4: “Poor communication” - FG5: “Patients not telling their family members (risk of AMD / genetics)” - FG5: “Lack of awareness of AMD in the community” - FG5: “Hard to balance communication / pitch from eye care practitioners (e.g. you can go blind versus it will take a while)” - FG5: “Language (NESB)” - FG5: “Timing of advice”   **Underutilisation of optometry**   - FG1: “No previous eye exam” - FG3: “Optometry not perceived as a carer in the health system” - FG3: “Perception that optometrists are ‘just spectacle providers’ – impact on access and believing advice” - FG5: “[Optoms] not wanting to ‘sell’ supplements” - FG6: “No eye tests (can use ready-made specs / magnifiers) more a problem in rural”   **Incurable disease**   - FG3: “Lack of evidence”   **Support**   - FG2: “Carer duties” - FG2: “Manage the amount of regular reviews, confusing for patients” - FG7: “’Sick spouse syndrome’”   **Injection**   - FG3: “Barriers of wet AMD (frequency of injections)” - FG4: “Fear (e.g. injections)” - FG7: “Patients don’t understand injections / expectations / some ophthalmologists don’t explain injections”   **Scope of practice**   - FG5: “Want glasses, not AMD chat, no smoking, no diet, stop smoking, etc.” - FG4: “Optoms not aware of best / latest treatment”   **Time**   - FG4: “Time (patient or carer does not have time)” - FG5: “Practitioner chair time tight” - FG7: “Lack of time (practitioner) – no time to do OCT / do injections anyway (in country) (referring to ophthals not always doing OCT on patients, rather just injecting on routine)”   **Care guidelines**   - FG3: “AREDS classification too hard for optometrists” - FG3: “[Lack of] clear guidelines (e.g. MD Foundation)” - FG6: “Not given choice / patient not involved in the care plan”   **Amsler grid**   - FG3: “Not enough use of Amsler grid (patients / practitioners)” - FG7: “Patients not using Amsler grid at home” - FG7: “Defects don’t show up on Amsler” - FG7: “Optoms / ophthal not explaining Amsler: benefits of daily check”   **Supplements**   - FG3: “Supplements too big to swallow” - FG5: “Conflict / confusion in MacuVision dosage (bottle versus what practitioner says)” - FG6: “Stop taking supplements after a while (understanding + cost)” - FG7: “Side effects of supplements (upset tummy / tablets). Patient don’t like to take supplements / can’t swallow”   **Miscellaneous**   - FG4: “No [anti]-smoking campaigns related to AMD” - FG7: “Hard to predict progression” | Structural  Patient-centered  Structural  Clinician-centered  Structural  Structural  Clinician-centered  Patient-centered  Patient-centered  Clinician-centered  Patient-centered  Patient-centered  Structural  Patient-centered  Patient-centered  Patient-centered  Clinician-centered  Structural  Structural  Clinician-centered  Patient-centered  Patient-centered  Patient-centered |

FG1 = Melbourne, VIC (Metropolitan); FG2 = Gold Coast, QLD (Metropolitan); FG3, FG4, FG5 = Sydney, NSW (Metropolitan); FG6 = Toowoomba, QLD (Regional); FG7 = Orange, NSW (Regional)
