# Supplementary material for: A qualitative exploration of Australian eyecare professional perspectives on Age-Related Macular Degeneration (AMD) care
Source: PLoS One. 2020 Feb 11;15(2):e0228858. doi: 10.1371/journal.pone.0228858 (PMC7012424; doi:10.1371/journal.pone.0228858)
Supplement: S4 Table — AMD care category themes and enablers to AMD care, category of influence and rank-ordering votes across three optometry focus groups. (DOCX) [file pone.0228858.s004.docx]

**S4 Table. AMD care category themes and enablers to AMD care, category of influence and rank-ordering votes across three optometry focus groups.**

| **Category themes and associated enablers nominated across focus groups** | **Category of influence** | **No of votes** | **Score** | **Rank ordering votes across groups** | **Total score** |
| --- | --- | --- | --- | --- | --- |
| **Education**   - FG2: “Start talking about LV earlier + other impacts (e.g. mobility, etc.). Promotion of Amsler grid & check both eyes. Greater / better education to patients earlier.” - FG6: “AMD education for optoms/GPs/dispensers/pharmacists/nurses/ophthalmologists” - FG1: “Education to GP/optoms on how to educate and how to treat” - FG1: “Involving family / carer when talking about disease” - FG1: “Educate children in school” - FG1: “App to educate and remind patients of Amsler grid ($65 USD)” | Patient-centered  Clinician-centered | 8  6  4  4  1  1 | 36  23  16  14  3  2 | Rank 1: 9  Rank 2: 6  Rank 3: 4  Rank 4: 5  Rank 5: 0 | 94 |
| **Access**   - FG1: “Better access to treatment / care including imaging (more money, training), equipment subsidies, care money and infrastructure money, reducing costs of care (medicare subsidies, OCT fees, anti-VEGF subsidy, supplement subsidy, mobile care unit to aged care facilities, travel allowance)” - FG6: “Robust public health system” - FG6: “[More] ophthalmologists who bulk bill” - FG2: “Shorter waiting list in public hospital system” - FG6: “More ophthalmologists in rural locations” | Structural | 15  3  2  2  1 | 49  11  10  3  1 | Rank 1: 5  Rank 2: 4  Rank 3: 7  Rank 4: 5  Rank 5: 2 | 74 |
| **Shared care model**   - FG2: “Flowchart & designated “in charge” practitioner & has to be paid (e.g. diabetes model). Psychological after care (needs to be on flowchart). Ongoing multidisciplinary follow-ups.” - FG2: “Encourage health practitioners to refer to a centralised spot (e.g. MD Foundation) - FG6: “Case manager (e.g. diabetes care plan)” - FG2: “Less variation between hospitals – clearer, more uniform pathways to hospital / local health services. Optoms & GPs need to understand their local hospital system and their role in it.” - FG2: “Easier referrals / less red tape to refer to Vision Australia or ophthalmology” - FG2: “Reports back from hospital” - FG1: “Psychological / motivational assessment” | Clinician-centered  Structural | 10  4  3  2  2  1  1 | 43  10  6  5  3  1  1 | Rank 1: 6  Rank 2: 2  Rank 3: 5  Rank 4: 6  Rank 5: 4 | 69 |
| **Communication**   - FG1: “Tailor communication to patient (e.g. more than pamphlet as LV can’t read, speaking to nurse or optom, materials in other languages)” - FG1: “Learn from other professions / fields about how to communicate e.g. amblyopia / aged-care / geriatric” - FG2: “Better communication between GP / optoms. More GP recognition of the potential role of optometry” - FG1: “Better tracking of follow-up appointments: follow-up appointments made and kept” | Clinician-centered  Patient-centered | 10  6  4  1 | 29  25  11  3 | Rank 1: 4  Rank 2: 4  Rank 3: 6  Rank 4: 5  Rank 5: 2 | 68 |
| **Funding**   - FG2: “Lower cost (subsidies for LV aid) e.g. private health care not always covering e.t. NDIS only 65 and under. Subsidise cost of treatment (e.g. injections) to allow private care.” - FG6: “NSID support for AMD > 65 years old, MyAgeCare” - FG6: “Being able to have OCT and perform it” - FG2: “Great medicare rebates for optometrists - FG1: “Fee for optometrist time to educate patients” | Structural | 8  4  2  2  1 | 28  10  8  4  4 | Rank 1: 0  Rank 2: 9  Rank 3: 4  Rank 4: 2  Rank 5: 2 | 54 |
| **Awareness**   - FG2: “Public awareness: need a media campaign on role of optometry. Media campaigns on success story” - FG6: “Advertisement awareness campaigns” - FG1: “Target over 60s during AMD week pamphlets, etc. (through GP)” - FG1: “Give old patients Amsler grids (with Senior’s card)” - FG1: “Community forums on AMD” - FG6: “Removal of stigma of low vision” | Patient-centered | 5  3  2  2  1  1 | 12  12  7  6  2  1 | Rank 1: 2  Rank 2: 2  Rank 3: 4  Rank 4: 4  Rank 5: 2 | 40 |
| **Low vision care**   - FG1: “Improve LV care” - FG2: “Improve optoms’ LV skills for general practice” - FG2: “Increase access to LV – more clinics or mobile clinics” - FG6: “Availability of LV function on technology” - FG6: “More facilities to try LV aids in rural locations” | Structural | 7  3  4  2  1 | 19  5  4  3  2 | Rank 1: 2  Rank 2: 3  Rank 3: 1  Rank 4: 2  Rank 5: 9 | 33 |
| **Research**   - FG1: “Keep researching AMD to find a cure – funding (research bionic eye)” - FG1: “KAP survey of patients and practitioners” | Structural | 13  2 | 28  3 | Rank 1: 2  Rank 2: 2  Rank 3: 0  Rank 4: 2  Rank 5: 9 | 31 |
| **Smoking / Food regulations**   - FG1: “Ban smoking or increase tax on smoking / bad food” | Patient-centered | 4 | 14 | Rank 1: 2  Rank 2: 0  Rank 3: 1  Rank 4: 0  Rank 5: 1 | 14 |
| **Genetic**   - FG6: “Genetics” | Patient-centered | 3 | 2 | Rank 1: 0  Rank 2: 0  Rank 3: 0  Rank 4: 1  Rank 5: 1 | 3 |

FG1 = Melbourne, VIC (Metropolitan); FG2 = Gold Coast, QLD (Metropolitan); FG6 = Toowoomba, QLD (Regional)
