# Supplementary material for: A qualitative exploration of Australian eyecare professional perspectives on Age-Related Macular Degeneration (AMD) care
Source: PLoS One. 2020 Feb 11;15(2):e0228858. doi: 10.1371/journal.pone.0228858 (PMC7012424; doi:10.1371/journal.pone.0228858)
Supplement: S5 Table — Enablers nominated by optometrists in at least one focus group but not selected during the “top five barriers” ranking process. (DOCX) [file pone.0228858.s005.docx]

**S5 Table. Enablers nominated by optometrists in at least one focus group but not selected during the “top five barriers” ranking process.**

| **Category themes and associated enablers nominated across focus groups** | **Category of influence** |
| --- | --- |
| **Education**   - FG1: “Primary care centres (e.g. diabetes) [should] give more information / suggest [AMD] assessment”   **Shared care model**   - FG1: “Liaise with home health (e.g. OT, council)”   **Communication**   - FG6: “MD Foundation material” - FG1: “Vision Australia app or app mentions Vision Australia”   **Funding**   - FG6: “Funding for LV aid / technology (government and private health)”   **Miscellaneous**   - FG6: “Trust between patients and optometrists” - FG6: “Improved treatment” - FG1: “Evidence for the patient that condition is under control (e.g. imaging, Amsler)” - FG2: “SMARTSight ≠ VIAOPTA and other such programs of structure patient program to keep taking injections” | Patient-centered  Structural  Patient-centered  Structural  Clinician-centered  Patient-centered |

FG1 = Melbourne, VIC (Metropolitan); FG2 = Gold Coast, QLD (Metropolitan); FG6 = Toowoomba, QLD (Regional)
