# Supplementary material for: A qualitative exploration of Australian eyecare professional perspectives on Age-Related Macular Degeneration (AMD) care
Source: PLoS One. 2020 Feb 11;15(2):e0228858. doi: 10.1371/journal.pone.0228858 (PMC7012424; doi:10.1371/journal.pone.0228858)
Supplement: Attachment — Submitted filename: Response to Reviewers.docx [file pone.0228858.s006.docx]

11 August 2019

Editor-in-Chief

PLOS ONE

Dear Editor,

Thank you for your review of our original manuscript PONE-D-18-34280 titled “*A qualitative exploration of eyecare professional perspectives on Age-Related Macular Degeneration (AMD) care*”. Please find below a table detailed our response to reviewers’ comments.

| **Academic Editor comments** | |
| --- | --- |
| This study used a qualitative approach to assess Australian eye care professionals approaches to patient care for those with Age-related macular degeneration (AMD). Both reviewers indicate that a qualitative approach is appropriate to assess this important area of health care, and that the study is interesting and important for improving care of patients with AMD. The Reviewers both raised methodological concerns, related to the structure of the interviews, the study sample included and the rationale for aspects of the interviews, focus groups and questions. Reviewer 2 in particular notes 3 main areas in general comments that should be addressed. These refer to a more detailed explanation of the study design, including rationale for using semi-standardised interviews, explorative interviews and focus groups; the application of findings beyond the groups interviewed, and clarification of the aims of the research project overall. | Thank you for your comments and this acknowledgement of the appropriateness and importance of this research. The reviewers’ concerns regarding the methodological concerns have been addressed as detailed in the Table below including a strong explanation of the rationale for combining explorative interviews and focus groups and appropriate clarification of the aims of the research. |
| As this is an Australian-based study, and the experiences for AMD patient care in health systems in other countries will be different, please consider including ‘Australian’ in the title | Thank you for the excellent suggestion. “Australian” has been added to the title of the manuscript. |
| Reviewer 2 also indicates that some details on epidemiology of AMD in Australia be included, as well as comments on any specific guidelines for AMD care in Australia. | References to the epidemiology of AMD outside of Australia as suggested by Reviewer 2 have been added. The following sentence was added to the beginning of the manuscript with appropriate citation to Wong et al. 2014 Lancet Global Health paper: “In 2013, 8.7% of the world’s population aged above 45 years had age-related macular degeneration (AMD) and the number of people affected is projected to reach 196 million by 2020 and 288 million by 2040 [1].  The following sentence was added to the introduction on page 4: “The lack of availability of evidence-based clinical care guidelines for AMD in many countries including Australia may also play a role [25, 26].” |
| **Reviewer 1 comments** | |
| An interesting study that adds to research and provides recommendations to improve AMD patient care. The quotes were useful and meaningful. The recommendations are useful for policy changes e.g., implementation of clear referral and care pathways, implementation of case managers (through changes in health funding) to communicate and facilitate optimal AMD care. The findings of this study may contribute to an improvement of quality of life in AMD patients and reducing the burden of this condition. The manuscript was well written and laid out. | Thank you for your positive overall comments on the study. |
| Materials and methods. Why did the semi-structured interviews range from 15 minutes to 60 minutes? Was there not a standard transcript? This needs to be explained / clarified. Perhaps include the transcript / topic guide in supps? | Thank you for the query. The description of timing in the manuscript was modified on page 6 to state that “interviews varied from 14 minutes to 33 minutes”. The topic guide has been included as suggested by the reviewer (new S1 Table 1). The numbering for other supplementary tables was adjusted accordingly (now S2 to S5). The interviews were semi-structured and flexible, and it is therefore not surprising that some participants may have had more to contribute in answer to the questions than others and therefore require a longer interview. |
| The two key questions asked in the focus groups – were they used in the individual interviews? Need to clarify (as in above point). | Yes, the questions used in the focus groups were also asked during the individual interviews, although the questions were broken down into several questions asked at different times during the semi-structured interview process, as shown in the semi-structured interview guide provided (new S1 Table). |
| For the focus groups – did the 5 minutes brief at the start of the focus group prime the focus group participants? It may have been useful to facilitate focus groups without this preamble to see if the responses were the same. How do you know if these 5 minutes brief introductions influenced participant responses? Overall, this introduction highlighted the adherence to recommendations being less than adequate, which may have made participants’ defensive about their profession or their own professional practice. This should be highlighted perhaps as a limitation. | The researchers agree with reviewer that this is a limitation of the study design. The following has been added to the discussion on page 29: “The brief presentation at the opening of the focus group may have influenced participant responses.” |
| The focus groups had two subgroups (the barriers groups and the barriers plus enablers groups) – this didn’t seem to be the case in the interviews i.e., they were all barriers plus enablers – is that correct? Did you examine if the responses from the two subgroups (compare the two subsets of focus groups) and the individual interviews were the same? If you compared groups, this could be specified. | Yes, this is correct. At the reviewer suggestion, the researchers examined these data and the following statement was added to the manuscript on page 16: “When responses of focus groups and interviews where participants discussed both barriers and enablers to AMD care were compared to those focus groups where participants discussed barriers only, no clear patterns or differences could be detected.” |
| Results. Why do you think there were such variations in the number of barriers in Table 2 eg for 15 participants, one group identified 20 where another group identified 38? To discuss. | Thank you for drawing attention to this interesting variability. The following was added to the discussion on pages 28-29: “The number of barriers identified by different focus groups varied from 18 to 38 (Table 2). This variability perhaps reflects the characteristics inherent to focus groups conducted with the nominal group technique: those where a low number of barriers was identified may have involved a dominant participant whereas those focus groups where a higher number of barriers were generated may have involved participants with more diverse views. The level of homogeneity present in the participants involved in each focus group (e.g. age, gender) may also have influenced these findings.” |
| Your theme of understanding/denial (as explained in S1 Table) seems to include many different / somewhat unrelated themes – for example lack of understanding of AMD and the impact it has on everyday life (e.g., due to poor communication) is a different concept to denial (not wanting to acknowledge or accept they have a chronic, progressive disease), which is again different to fear of injections. To be addressed.  There is a lot of cross-over between the care pathway theme and the underutilisation of optometry theme as indicated by the examples in Table S1. Can the differences between these themes be made clearer?  Also, what is the difference between care pathway and care guidelines in Table S1? | Thank you for this comment. S1 Table is now S2 Table in the revised version. The following was added to the Results section of the manuscript on pages 15-16 in response to this and a similar comment from Reviewer 2: “The amalgamation of barriers involved much discussion by participants during the focus group consensus building exercise as well as by researchers involved in the iterative thematic analysis. Where full agreement could not always be reached, a compromise position may have been adopted. As a result, readers may perceive similarities between barriers that were not amalgamated and distinctions between those that were. Areas of potential cross-over are discussed in more details here. The theme understanding/denial is a broad theme that was amalgamated from 16 identical and/or related barriers identified and described by focus group participants. These barriers encompassed concepts related to denial and fear (including fear of injection), mental state, lack of understanding, complacency, and lack of knowledge. Central to this theme was the notion that AMD is often asymptomatic at time of diagnosis. Many discussions during focus groups centered around the consequences of this lack of symptoms on people with AMD’s understanding and belief regarding their disease and how this could combine with fear to enable denial. The poor care pathway theme centered around the complexity, and lack of clarify of the journey back and forth to and between services and providers for people with AMD. This went beyond optometry or ophthalmology to include general practitioners, dieticians, low vision and rehabilitation services providers and even included discussion of transport subsidies and case managers. The underutilisation of optometry theme although related centered around the role of optometry as a primary care provider that can screen, detect, triage and direct people with AMD to relevant services. The care guidelines theme focused not on the journey between service providers back rather on the lack of availability of evidence-based clinical care guidelines and the resulting lack of clarity as to what the best or most appropriate treatment might be for people with AMD.” |
| The two subgroups could be defined more clearly in the text and a footnote could be included about the differences between these groups in Table 2 (to make it clearer why there are blank squares in the table). | Thank you for the suggestion. The following sentence was added to the Methods section on page 7: “Barriers to AMD care were generated by all seven focus groups whilst enablers to AMD care were generated in a subset of three out of seven focus groups only.”  A footnote to the same effect was added to Table 2 (page 11) as suggested by the reviewer. |
| Figure 1 and Figure 2 – if possible should include the barriers and enablers in the figure to make them easier to differentiate. | To ensure readability of the Figure and as each Figure already contains a significant amount of data (dual x-axis, 7 data series), we prefer to retain these in separate Figures. |
| Is the first quote supposed to say ophthalmologist not ophthalmology as the final word? | Thank you this was corrected. |
| S3 Table has NSID instead of NDIS. | S1 Table is now S2 Table in the revised version. This has been corrected. |
| As in line 291, the first reference to “enablers” in text should be described as “enablers of good AMD care” | Page 14 line 298 now states that “Fifty-one enablers of appropriate AMD care were identified …..” |
| **Reviewer 2 comments** |  |
| The manuscript deals with an important topic: the challenges of providing adequate, evidence-based care for patients with age-related macular degeneration. Choosing a qualitative study design is legitimate, as many of the barriers for AMD management in everyday care are not well understood, so an explorative approach is sensible. However, there are some significant methodological concerns with the study presented. | Thank you for your comments and providing your expertise to this review. In the revised manuscript, efforts have been made to address, explain, justify and/or recognise any of the methodological concerns highlighted by both reviewers more fully as described above and below. |
| First, the study design is not satisfying. The authors do not only combine semi-standardised interviews and focus groups, which would have been acceptable if the rationale was explained properly. The authors combine explorative interviews with ophthalmologists on the one hand, with a semi-quantitative consensus approach with optometrists on the other hand, and the research questions in both data collections are different as well: whereas the interviews with ophthalmologists covered AMD referral pathways, communication, compliance and sources of information, the nominal group technique with optometrists covered, “under and over detection and inappropriate AMD management”. Why the authors chose to focus on different aspects in the different samples is not explained. I believe very well that the authors had difficulty recruiting clinicians for focus groups, but if they convert to interviews within this sample, they should at least use the same research questions in both samples, and follow the same study logic (either explorative or consensus-oriented). | Thank you for these important comments. The aim of the study was clarified to state: “The current study aimed to explore the perspectives of eyecare professionals (optometrists, ophthalmologists) on AMD care in Australia, with a view to identify the most important barriers and enablers to appropriate AMD care and through this describe some of the possible reasons for under or over detection and inappropriate management.”  The approach taken for this research combining explorative and consensus-oriented methods is not unique (see Tong A et al for the SONG-Kids studies, Trials 2016; 17: 401). However, the presentation of the rationale for the combined methods approach allowing triangulation of the data was strengthened in the manuscript and the limitation of the pragmatic approach taken to ophthalmology sampling was highlighted more strongly in the discussion as follows.  A statement was added to the Materials and Methods on page 5: “The original intent was for this research to adopt a multimethod approach which combined several methods to collect information on a given topic [28]. […] In this research, semi-structured interviews and focus groups were conducted in parallel and combined in a process known as method triangulation where there was mutual enhancement of the understanding of barriers and enablers to AMD care of each method by the other.”  The researchers focused on the same questions in both samples. The relationship between the research questions used in each method was clarified by:   - combining the two aim statements as “The semi-structured and focus groups specifically aimed to identify the most important barriers and enablers to optimal AMD care. The semi-structured interviews allowed for more detailed exploration of additional aspects to be undertaken” on page 6. - Providing a copy of the final semi-structured interview guide as Supplementary Table 1.   And add to the discussion on page 28: “The concurrent use of an explorative and consensus-oriented method allowed both qualitative and semi-quantitative data to be collected, inform and complement each other but the lack of ophthalmology involvement in the focus group aspect of this research is a limitation [28].”  And on page 29: “Recruitment difficulties prevented the conduct of planned ophthalmology focus groups; the semi-quantitative findings presented in this study may not represent the ophthalmology perspective.” |
| Second, qualitative methods can be an excellent tool to explore a person’s experiences, fears, motives, practices etc, so as to better understand this person’s behaviours or decisions. Therefore, they can generate first-hand evidence on reasons behind phenomena. One should be very cautious, however, in using qualitative data collection (or quantitative, for that matter) for exploring motives of people other than the interview partners. The main questions asked of the optometrists in the focus groups refer to “factors preventing people at risk of or with AMD from accessing and/or receiving good care and/or from following advice given to them”. The answers to this question can only be speculation; one would need to ask patients if one is interested in understanding what is preventing them from accessing care, or adhering to recommendations. | Thank you for highlighting this limitation of the study. This is already somewhat acknowledged in the manuscript on page 29 with the statement “This study’s findings would ~~also~~ therefore benefit from cross-tabulation with the perspective of people with AMD and their carers. This research has been conducted and will be reported separately.”  The following sentence was added to the same paragraph on page 29 to more clearly acknowledge this limitation: “The findings reported in this study represent the perspective of eyecare professionals on a research question that also involves people with AMD and their access to care; eyecare professionals’ perspective of the motives and behaviour of people with AMD should be interpreted with caution.” |
| Third, the research question needs to be focused, or the introduction changed. Whereas the introduction deals almost exclusively with lifestyle risks and preventive counselling in patients with AMD, the research question is much broader – it refer to “AMD care in Australia”, including “under or over detection and inappropriate management”. This would also include screening, diagnostic means, drug therapy (anti VEGF injections), check ups, none of which is even mentioned in the introduction. In the methods section, also “referral pathways / access to care” are named as topics, and the main question in the nominal group technique is patient’s adherence to recommendations. The literature existing on patients reasons for non-adhering to AMD therapy is not mentioned in the manuscript. Also, whereas focus groups and interviews are meant to explore a wide range of perspectives, nominal group technique is about consensus and effective decision-making. What was the aim of the research – to identify a consensus / ranking among eye care professionals on the most important barriers / facilitators, or was it about eliciting a wide range of barriers present in eye care? -- | The aim of the study was clarified and the justification for combining the nominal group technique and explorative methods provided as described above.  As the aim is broad i.e. “To explore the perspectives of eyecare professionals on AMD care in Australia with a view to identify the most important barriers and enablers to appropriate AMD care”, the introduction was modified to include additional concepts as suggested by the reviewer, This was achieved by adding on page 3: “For neovascular AMD, effective treatments that can slow the progression of disease are available in the form of anti-VEGF infections [6].” and on page 5 “The lack of availability of evidence-based clinical care guidelines for AMD in many countries including Australia may also play a role [25, 26].” In addition, “poor awareness, professional development” were added to the existing list of known barriers on line 84. |
| Specific comments |  |
| Introduction:  Please include some AMD prevalence data of other countries to highlight the relevance of the disease for readers outside Australia (high prevalence rates can indeed be found in all industrialized countries). | Thank you for the suggestion. The following sentence was added to the beginning of the manuscript with appropriate citation to Wong et al. 2014 Lancet Global Health paper: “In 2013, 8.7% of the world’s population aged above 45 years had age-related macular degeneration (AMD) and the number of people affected is projected to reach 196 million by 2020 and 288 million by 2014 [1]. |
| p.2 l.33: “Clear, evidence-based recommendations on appropriate modifications to recommend for people”: It would be important to mention in which form these recommendations are available. Are there clinical practice guidelines on AMD available in Australia? The content of guidelines from US-America, NICE (UK), Germany and other countries differ from each other in terms of what lifestyle and nutrition advice should be given to patients and are not always “clear”. | This is an excellent comment and we agree that availability, clarity, and dissemination of clinical practice guidelines on AMD has not always been optimal. On that basis, the statement has been modified and narrowed, and appropriate references to more adequately reflect the reality of practice were added as follows: “~~Clear,~~ Evidence-based recommendations on appropriate modifications to recommend for people with and/or at risk of AMD have existed for some years [4, 9], yet self-reported practices suggest that eyecare professionals’ advice to their patients on smoking cessation and dietary supplementation are often lacking [10-12]. |
| As barriers are the core issue of study, it would helpful to include more information on barrier research or systematic taxonomies of barriers available in the literature, e.g. the framework by Cabana et al (“Why don’t physicians follow clinical practice guidelines? A framework for improvement.” JAMA. 199; 282: 1458-65) or the well-tested theoretical domains framework, see e.g. Cane et al. Validation of the theoretical domains framework for use in behaviour change and implementation research. Implement Sci 2012; 7, or Birken et al. Combined use of the Consolidated Framework for Implementation Research (CFIR) and the Theoretical Domains Framework (TDF): a systematic review. Implement Sci 2017; 12) | Thank you for this excellent suggestion; this work was planned and has already commenced. A paragraph discussing the published frameworks available to conceptualise barriers and access to health care has been added to the discussion on page 30 as follows: “Significant research has been conducted to understand the barriers to use of clinical practice guidelines and evidence-based practice by health professionals [18, 57, 58] and/or barriers to patients’ access to care [19]. From this research a number of theories, models and frameworks including the well-known theoretical domains framework (TDF) [57] are available that can be applied to research such as that presented in the current study to help understand, explain, and address the gaps between research and practice in the area of AMD care. Following the identification of the determinants of AMD care (i.e. barriers and enablers) in this study, application of one or several of these frameworks to the study findings could help to refine and confirm the appropriateness of the recommendations suggested below. This work is currently underway and will be reported separately.” |
| Methods:  The authors claimed they used purposive sampling and “maximum variation” in the sample – but the sample was obviously not generated in a purposive way, as none of the members worked in rural settings, and all ophthalmologists worked in metropolitan areas. So it seems as though the authors followed a convenience sampling and then characterized the sample according to the practice setting, instead of deliberately recruiting participants from different locations. | Thank you for the comment. Although purposive sampling was attempted in this research, the reviewer is correct in that because of recruitment difficulties the final pragmatic approach to recruitment that was adopted is best described as convenience sampling. This was clarified in the manuscript by stating that purposive, snowball, and maximum variation sampling were “planned” (instead of “used”) and by adding the following statement on page 6: “As a result, the sampling method for this study is best described as convenience sampling, recognising that efforts were made to ensure appropriate representation where possible.” |
| It is not clear whether the focus groups were indeed performed as focus groups with questions alongside a guide or a list of prompts, or whether they merely consisted of the nominal group technique during which the participants’ commentaries were audiotaped. As mentioned above, focus groups are explorative by nature, whereas NGT is consensus-oriented. | The reviewer is correct. The focus groups were conducted using the nominal group technique using a consensus-oriented approach as currently described in the manuscript. The approach used in this research where explorative and consensus-oriented methods were combined was made clearer and a rationale provided as described above. |
| p. 6, l.120: “compliance to evidence-based care”: Whose compliance – health care professionals compliance to guidelines, or patients’ compliance to recommendations? | Compliance of both health care professionals and patients was explored during the semi-structured interviews. This was added to the manuscript on page 6. The interview guide is now provided as S1 Table. |
| The description of the private services that participants worked with (EyecarePlus, ProVision, EyeQ or SpecSavers, OPSM, Bupa) is probably not comprehensible to readers outside Australia. | Thank you for this comment. These examples were removed from the manuscript as it is not feasible to provide examples for individual countries outside of Australia. |
| Results:  In the “Overview” (p. 11 and following), the barriers and facilitators identified in the NGT are not properly explained, just mentioned as brief terms such as “Amsler grids”, “communication” etc. For example, what kind of barrier is “lifestyle changes”? what is meant with “Understanding/denial” – patient understanding of disease? Optometrists understanding of (conflicting, changing) evidence? Denial of being ill – denial of effectiveness of prevention? | The following was added to the Results section of the manuscript on page 16 in response to both this comment and a similar comment from Reviewer 1: “The theme understanding/denial is a broad theme that was amalgamated from 16 identical and/or related barriers identified and described by focus group participants. These barriers encompassed concepts related to denial and fear (including fear of injection), mental state, lack of understanding, complacency, and lack of knowledge. Central to this theme was the notion that AMD is often asymptomatic at time of diagnosis. Many discussions during focus groups centered around the consequences of this lack of symptoms on people with AMD’s understanding and belief regarding their disease and how this could combine with fear to enable denial. The poor care pathway theme centered around the complexity, and lack of clarify of the journey back and forth to and between services and providers for people with AMD. This went beyond optometry or ophthalmology to include general practitioners, dieticians, low vision and rehabilitation services providers and even included discussion of transport subsidies and case managers. The underutilisation of optometry theme although related centered around the role of optometry as a primary care provider that can screen, detect, triage and direct people with AMD to relevant services. The care guidelines theme focused not on the journey between service providers back rather on the lack of availability of evidence-based clinical care guidelines and the resulting lack of clarity as to what the best or most appropriate treatment might be for people with AMD.” |
| The results of the explorative section are not very convincing. The results touch rather superficially at a very broad range of topics – competition between optometrists and ophthalmologist, difficulties related to preventative counselling, difficulties in access to care in old people with impaired vision. For example, a survey among ca. 900 AMD patients and 900 caregivers in different countries (among others, Australia), identified a number of barriers preventing patients from accessing AMD care (Varano et al, Clin Ophthalmology 2015). Given this existing comprehensive evidence, it is not really a novel finding if optometrist comment on transport problems of AMD patients. In addition, some important aspects are only illustrated by quotes of optometrists (e.g. Referral pathways and models of care, perception and awareness of clinical roles), which does not seem very balanced given the topic. | As described in the manuscript, topics highlighted in the explorative section of the results were selected based on their importance from the perspective of the eyecare professionals that participated in the study (highly ranked in the nominal group technique and/or number of cites in the qualitative analysis). We acknowledge that other researchers have previously identified many of these barriers to care but our mixed-method approach provides more depth to these findings. This was clarified by adding the words “(based on rank and cites)” on page 12.  The quotes were selected based on their ability to best highlight the themes discussed and not based on the health professional type and this is appropriate. An ophthalmology quote has been added to balance the presentation of the Referral pathways and models of care, perception and awareness of clinical roles theme as follows on page 19: “Again, it probably reflects dealing with the public hospital workload, it’s only going to be possible for us to manage what we’re doing in public hospitals by having optometrists dealing with early stages of macular degeneration and being able to explain to the patients well and not cause them worry.” Ophthalmologist P54. |
| Discussion:  p.22, l 490-491: “Also lacking in the AMD care pathway is a recognition of the role of support services (e.g. mobility 491 and orientation, dietician, counselling, etc.)”.: I could not find data on this aspect in the results. On the contrary, the participating eye care professionals stated that they consider diet etc important, but patient response to counselling, or funding of counselling, is not satisfying. | This statement has been removed from the discussion. |
| P.23 l 515-6: “Clinicians’ skills and knowledge and misdiagnosis were identified as significant barriers to good AMD care in this study.” I could not find any statement in the results pertaining to misdiagnosis as a barrier. Interviewed ophthalmologists suspected other ophthalmologists to be not as skilled as they were themselves, and to offer IVOM treatment only for monetary reasons. But in fact, as it is only ophthalmologists making speculations about potential competitors, I think it is dangerous to deduct from these statement the assumption that many clinicians are not skilled. | The reviewer is correct. This was addressed by removing the word “misdiagnosis” on page2 21 and 25 so that the focus of the discussion returns to skills and knowledge as is present in the results. |

I thank you in advance for your consideration and the reviewers for their substantial contribution to this manuscript.

**Isabelle Jalbert, OD, PhD, MPH, GradCertOcTher, FAAO**

Associate Professor, Deputy Head of School

School of Optometry and Vision Science

Associate Dean, International and Engagement, Faculty of Science

Email: [i.jalbert@unsw.edu.au](mailto:i.jalbert@unsw.edu.au)
